# Anomalous Origin of the Right Coronary Artery from the Pulmonary Artery (ARCAPA) in Adults: Analysis of 59 Clinical Cases

**DOI:** 10.3390/jcm15145372

**Published:** 2026-07-09

**Authors:** Kristina Gennadievna Pereverzeva, Ekaterina Alekseevna Smetanina

**Affiliations:** Department of Hospital Therapy, Ryazan State Medical University, 390000 Ryazan, Russia; katya.smetanina2@yandex.ru

**Keywords:** ARCAPA, anomalous origin of the right coronary artery from the pulmonary artery, surgical correction, conservative management, myocardial ischemia

## Abstract

**Background:** Anomalous origin of the right coronary artery from the pulmonary artery (ARCAPA) is an extremely rare and potentially fatal congenital heart defect, accounting for approximately 0.003% of all coronary artery anomalies. **Methods:** A literature search was performed in the PubMed and eLibrary databases using the keywords “ARCAPA” and “anomalous origin of the right coronary artery from the pulmonary artery” for the period from 2005 to 2025. A total of 158 papers were screened, from which 57 articles reporting 59 clinical cases in adult patients were selected. **Results:** The median age was 55 years [18–80]. Males accounted for 54.2% (32/59). ARCAPA was an incidental finding during evaluation for another condition in 35.9% (21/59) of cases. Clinical presentation: retrosternal pain—47.5% (28/59), dyspnea—44.1% (26/59), and reduced exercise tolerance—35.9% (21/59). A completely asymptomatic course was observed in 22.0% (13/59) of patients. In rare cases, the initial presenting manifestations included atrial fibrillation, cardiac arrest during a marathon, and worsening dyspnea during pregnancy. The most commonly used diagnostic modalities were computed tomography angiography—in 67.8% (40/59) of cases—and coronary angiography—in 49.2% (29/59) of cases. Cardiac catheterization confirmed the diagnosis in only 10.2% (6/59) of patients. Surgical correction was performed in 54.2% (32/59) of patients; the most frequently used technique was reimplantation of the right coronary artery into the ascending aorta—in 42.4% (25/59) of cases. One long-term complication was recorded—right coronary artery thrombosis 17 years after surgery—and one case of sudden cardiac death occurred in an unoperated patient. Conservative management was chosen in 40.7% (24/59) of patients, mainly due to an asymptomatic course, high surgical risk, or patient refusal. **Conclusions:** ARCAPA is a rare anomaly for which surgical treatment appears to be the preferred approach in symptomatic patients or those with documented ischemia, while conservative management may be acceptable in selected asymptomatic patients. Conservative management is acceptable in truly asymptomatic patients, but regular follow-up is recommended.

## 1. Introduction

Anomalous origin of the right coronary artery from the pulmonary artery (ARCAPA) is a rare congenital anomaly first described by Brooks in 1885 [1]. Unlike its more well-known and prognostically unfavorable counterpart—anomalous origin of the left coronary artery from the pulmonary artery (ALCAPA), which leads to critical myocardial ischemia early in infancy—ARCAPA often has a more benign course and is frequently diagnosed incidentally in adulthood [2,3]. This delayed diagnosis is explained by the fact that in ALCAPA, perfusion of the large left ventricular territory is compromised immediately after the drop in pulmonary vascular resistance at birth, whereas in ARCAPA, a smaller territory is affected. In addition, given the well-developed collateral network from the left coronary artery (LCA) system, the perfusion deficit can be compensated for over a long period.

The pathophysiological basis of ARCAPA clinical manifestations is the phenomenon of coronary steal. Since pressure in the pulmonary artery (PA) is significantly lower than in the aorta, blood from the normally originating LCA system is shunted into the PA through well-developed intercoronary collaterals via the orifice of the anomalous right coronary artery (RCA). This pathological shunt leads to chronic myocardial hypoperfusion, which can cause ischemia, dilation and aneurysmal expansion of the coronary bed, myocardial dysfunction, heart failure (HF), life-threatening arrhythmias, and sudden cardiac death. It is important to note that the magnitude of this shunt varies depending on the pressure gradient between the aorta and the PA, as well as on the resistance of the collateral network. This fact explains the wide spectrum of ARCAPA clinical manifestations—from complete compensation to severe circulatory decompensation.

The clinical presentation of ARCAPA is highly variable: from a complete absence of symptoms throughout life to manifestation in adulthood with angina pectoris, HF, or sudden cardiac arrest. Rare presenting manifestations described in the literature include atrial fibrillation with a rapid ventricular rate and hemodynamic instability [4], cardiogenic shock due to acute right ventricular failure [5], cardiac arrest during a marathon in a previously asymptomatic young patient [6], and worsening dyspnea during pregnancy [7]. Such variability, together with the casuistic rarity of the defect, creates significant diagnostic difficulties and debates regarding the optimal management strategy for patients.

Despite the publication of many individual case reports, a comprehensive analysis summarizing current data on the demographics, clinical features, diagnosis, and treatment of ARCAPA is lacking. This study aims to fill this gap by conducting a narrative analysis of 59 available clinical cases reported in the world literature.

## 2. Materials and Methods

This is a narrative review with a systematic literature search, rather than a formal systematic review or meta-analysis. We did not register a protocol, perform formal quality assessment, or follow PRISMA guidelines, as these are not applicable to narrative reviews.

### 2.1. Literature Search Strategy

A literature search was conducted in two electronic databases: PubMed (U.S. National Library of Medicine) and eLibrary (Russian Scientific Electronic Library). The search covered the period from 1 January 2005 to 13 May 2025. We deliberately restricted the time frame to the past two decades in order to focus on studies that used contemporary diagnostic modalities (multidetector computed tomography angiography, cardiac magnetic resonance imaging, and advanced echocardiographic techniques) and to reflect current management paradigms for adult congenital heart disease. Earlier publications were excluded because they often lacked detailed imaging data and relied on diagnostic methods that are now obsolete.

The following search syntax was used in PubMed: (“anomalous origin of the right coronary artery from the pulmonary artery” [Title/Abstract] OR “ARCAPA” [Title/Abstract]).

In eLibrary, the search was performed using the Russian-language equivalents of the keywords: the Russian translation of “anomalous origin of the right coronary artery from the pulmonary artery” and the abbreviation “ARCAPA”. Only articles published in English or Russian were considered for inclusion. The reference lists of all retrieved articles were also manually screened to identify additional relevant publications.

### 2.2. Eligibility Criteria

1. Studies were eligible for inclusion if they met all of the following criteria:
2. Reported original clinical cases or case series of ARCAPA in patients aged ≥ 18 years;
3. Provided sufficient clinical, diagnostic, or treatment data to allow meaningful extraction;
4. Were published in English or Russian between 2005 and 2025;
5. Represented original reports (case reports, case series).

### 2.3. Exclusion Criteria

1. Patients < 18 years of age;
2. Non-original reports (reviews, editorials, letters to the editor, and comments without new data);
3. Duplicate publications (overlapping patient populations from the same institution);
4. Insufficient clinical information to extract relevant data;
5. Full text unavailable in English or Russian.

### 2.4. Study Selection Process

Study selection was performed in two stages. First, all titles and abstracts retrieved by the search were screened for relevance by one reviewer (E.A.S.). Potentially eligible full-text articles were then assessed independently by two reviewers (E.A.S. and K.G.P.) against the predefined inclusion criteria. Disagreements between reviewers were resolved by consensus.

A total of 158 records were identified through the database search. After screening titles and abstracts, 89 records were excluded (not original case reports, reviews, studies in patients < 18 years, or publications not in English or Russian). The remaining 69 full-text articles were assessed for eligibility, of which 12 were excluded (insufficient clinical data, full text unavailable, or overlapping patient populations). Thus, 57 articles describing 59 clinical cases were included in the final analysis [4,5,6,7,8,9,10,11,12,13,14,15,16,17,18,19,20,21,22,23,24,25,26,27,28,29,30,31,32,33,34,35,36,37,38,39,40,41,42,43,44,45,46,47,48,49,50,51,52,53,54,55,56,57,58,59,60].

### 2.5. Data Extraction and Synthesis

Data extraction was performed using a standardized form and included: patient demographics (age, sex), clinical presentation (symptoms, incidental finding, and unusual presentations), diagnostic imaging methods (electrocardiography, echocardiography, computed tomography angiography, coronary angiography, cardiac magnetic resonance imaging, myocardial perfusion scintigraphy, and cardiac catheterization), treatment details (surgical correction vs. conservative management, specific surgical techniques, and reasons for conservative management), and clinical outcomes (follow-up data, complications, and mortality). Data were extracted by one reviewer (E.A.S.) and verified by a second reviewer (K.G.P.).

### 2.6. Statistical Analysis

Statistical analysis was performed using Microsoft Excel (version 16.0, Microsoft Corp., Redmond, WA, USA). Quantitative variables were described using median (Me) and lower and upper quartiles [Q1; Q3] or range (min–max). Categorical variables were described using absolute numbers and percentages, with numerators and denominators explicitly reported wherever percentages are given (e.g., *n*/*N* [%]). Only patients with available data were included in the calculation of each parameter. No formal statistical hypothesis testing was performed due to the descriptive nature of the review, the small sample sizes, and the substantial amount of missing data across published case reports.

### 2.7. Nature of the Review

It is important to emphasize that this review is intended to provide a descriptive synthesis of published clinical experience with ARCAPA in adults. It does not aim to estimate disease prevalence, diagnostic test accuracy, or comparative treatment efficacy. All frequency data presented herein are derived from published case reports and case series and should not be interpreted as prevalence estimates or as direct measures of treatment effectiveness. The descriptive statistics summarize published experience rather than estimate population frequencies.

## 3. Results

A total of 59 observations of ARCAPA in patients over 18 years of age were included in the analysis. The median age was 55 [18–80] years, and males accounted for 54.2% (32/59). The key demographic, clinical, diagnostic, treatment, and outcome data for the overall cohort, as well as separately for symptomatic and asymptomatic patients, are summarized in Table 1.

It should be noted that all subgroup comparisons (symptomatic vs. asymptomatic, surgical vs. conservative) are purely descriptive. Formal statistical testing was not performed due to the small sample sizes and the substantial amount of missing data across published case reports. Therefore, these comparisons should not be interpreted as evidence of superiority of one management strategy over another.

ARCAPA was an incidental finding during evaluation for another condition in 35.6% (21/59) of cases (e.g., pneumonia, preoperative workup, oncological screening, or active tuberculosis) [38,55,56]. Some form of symptomatology was present in 78.0% (46/59) of patients, while 22.0% (13/59) were completely asymptomatic. Among the 46 symptomatic patients, the most common manifestations were: chest pain—60.9% (28/46); dyspnea—56.5% (26/46); reduced exercise tolerance—45.7% (21/46); palpitations—23.9% (11/46); syncope—4.3% (2/46). Weakness was reported in 5 patients (10.9% of symptomatic cases). In one case, pain radiated to the left arm and shoulder, and in another case, pain was accompanied by sweating and vomiting [54,57].

Several cases deserve special mention due to unusual initial presentations:

Atrial fibrillation: In one patient, ARCAPA first presented as atrial fibrillation with a high ventricular rate and hemodynamic instability requiring electrical cardioversion [4]; the arrhythmia recurred despite antiarrhythmic therapy and resolved only after surgical correction.

Cardiogenic shock: A patient with tachycardic atrial fibrillation developed acute right ventricular failure and cardiogenic shock; comprehensive imaging revealed ARCAPA as the underlying cause [5].

Cardiac arrest during a marathon: A 33-year-old previously asymptomatic marathon runner suffered out-of-hospital cardiac arrest at the 20 km mark of a race. Post-resuscitation computed tomography angiography (CTA) showed ARCAPA with well-developed collaterals but no significant coronary atherosclerosis [6].

Worsening during pregnancy: A woman with a known diagnosis of ARCAPA experienced progressive dyspnea in the second trimester of pregnancy, which the authors attribute to the physiological increase in pulmonary blood flow and cardiac output during pregnancy [7].

Active tuberculosis: In a patient with active pulmonary tuberculosis, ARCAPA was discovered incidentally; the infectious process precluded immediate surgical treatment, and conservative management was chosen [38].

Concomitant coronary fistula: In one case, ARCAPA was combined with a coronary fistula between the right coronary artery (RCA) and the left anterior descending artery (LAD), requiring extended preoperative planning [39].

Among the 46 symptomatic patients, the most common triggers for symptoms were physical exertion—in 23.9% (11/46); in 4.3% (2/46), cardiac symptoms began after opioid use [10,51]. In one patient, disease onset was registered during a hemodialysis session [27].

Laboratory data often remained within normal limits. Troponin was measured in 22.0% (13/59) of patients. Elevated levels were found in 7 of these 13 patients (53.8%), accounting for 11.9% (7/59) of the total cohort. Elevated troponin was associated either with acute coronary events or with other acute conditions (e.g., pulmonary embolism) [17,19,41,51,52,54]. These findings suggest that myocardial ischemia in ARCAPA is mostly subclinical and chronic in nature.

Echocardiography often served as the first method to raise suspicion of the diagnosis. Indirect signs detected by echocardiography included dilation and tortuosity of the coronary arteries (both right and left), abnormal color Doppler signals in the right ventricle indicating blood shunting into the PA, and signs of a coronary–pulmonary fistula [11,16,29,37,47,56]. However, direct visualization of the anomalous coronary ostium by transthoracic echocardiography is difficult and was successful in only 6.8% (4/59) of patients [20,50,59].

Computed tomography angiography (CTA) was performed in 67.8% (40/59) of cases. This CTA-based approach has largely replaced invasive coronary angiography (CAG) for initial diagnosis due to its high spatial resolution and lower risk. This method allows high-precision visualization of the anomalous origin of the RCA from the PA, assessment of coronary artery diameter, identification of the presence and extent of collateral circulation, and exclusion of other concomitant pathology [6,10,18,19,20,21,23,29,30,44,45]. This study was particularly valuable for cardiac surgeons, who used its results for preoperative planning. Coronary angiography (CAG) was performed in 49.2% (29/59) of cases. The pathognomonic angiographic sign of ARCAPA is retrograde contrast filling of the trunk and distal branches of the RCA through well-developed intercoronary collaterals (most often from the LCA system) followed by drainage of contrast into the pulmonary trunk. Cardiac magnetic resonance imaging (MRI) for anatomical assessment and differential diagnosis was performed in 11.9% (7/59) of cases. Myocardial perfusion scintigraphy (SPECT) was used to verify myocardial ischemia in the RCA territory and to evaluate postoperative treatment outcomes in 8.5% (5/59) of patients [17,34,47,54,55].

A significant number of patients had various cardiovascular risk factors: arterial hypertension in 32.2% (19/59), hyperlipidemia in 16.9% (10/59), and diabetes mellitus in 11.9% (7/59). Several patients had concomitant cardiac diseases (aortic valve stenosis, severe mitral regurgitation, coronary artery disease, atrial fibrillation). VACTERL association (vertebral anomalies, anal atresia, cardiac defects, tracheoesophageal fistula, esophageal atresia, renal anomalies, limb abnormalities) was a congenital background condition in one patient [9]. In one described case, ARCAPA was combined with a coronary fistula between the RCA and the LAD [39].

Surgical correction was performed in 54.2% (32/59) of patients. The most common and physiologically justified method was reimplantation of the RCA into the ascending aorta—in 78.1% (25/32) of cases. In some cases, coronary artery bypass grafting (CABG) with ligation of the RCA was used—15.6% (5/32)—and simple ligation of the RCA—6.3% (2/32). The choice of ligation without revascularization was a forced decision in patients with high risk of cardiopulmonary bypass, provided that collateral flow from the LCA was deemed adequate. Surgery was often combined with correction of concomitant pathology: valve replacement, mitral valve repair, resection of coronary artery aneurysms [13,44,58,59].

Conservative management and observation were chosen for 40.7% (24/59) of patients. Among the 24 patients managed conservatively, the reasons for this approach were: high surgical risk due to severe comorbidity (malignancy, pulmonary embolism, renal failure, decompensated HF, active tuberculosis)—29.2% (7/24); patient refusal of proposed surgical treatment—50.0% (12/24); and treatment still under consideration—20.8% (5/24), for whom appropriate medical support was temporarily provided. Medical therapy typically included standard medications used for chronic HF and coronary artery disease. In two clinical cases, no data on the treatment provided were available [23,30].

The vast majority of patients who underwent surgery demonstrated good immediate and long-term outcomes, with complete regression of symptoms, normalization of myocardial perfusion according to follow-up studies, and improvement in functional status [15,20,21,35,36,44,51,55,60]. One fatal outcome due to sudden cardiac death was reported in an unoperated patient with coronary artery disease; autopsy revealed ischemic changes in the myocardium of the left and right coronary artery territories, well-developed intercoronary collaterals characteristic of ARCAPA combined with severe atherosclerosis of the LCA, which, according to the authors, may have been the cause of death [26]. In another patient who underwent surgery 17 years earlier, RCA thrombosis and left circumflex artery stenosis were diagnosed and successfully stented; the authors note that the anomalous RCA does not tend to normalize in diameter after surgical correction, emphasizing the need for lifelong follow-up even after successful surgery [42].

## 4. Discussion

Our analysis of 59 clinical cases of ARCAPA in adults clarifies the key aspects of diagnosis and treatment of this rare anomaly. Given the narrative nature of this review, our findings are based on descriptive case data and should be interpreted with appropriate caution. In this discussion, we distinguish between descriptive data from our analysis, our interpretations, and recommendations based on published clinical guidelines.

It is also important to restate that this review is intended as a descriptive synthesis of published clinical experience, not as an estimate of disease prevalence, diagnostic test accuracy, or comparative treatment efficacy. All frequency data presented here are derived from published case reports and case series and should be interpreted with appropriate caution.

In our cohort, the median age was 55 years [18–80], and the rate of incidental detection was 35.6% (21/59). These data suggest that ARCAPA can have a long asymptomatic or oligosymptomatic course and first manifest in adulthood [5,14,17,28,39,43,49]. Our analysis also shows a wide variability of symptoms, which precludes a specific symptom complex and makes diagnosis difficult without targeted imaging [4,6,19,26,38].

Importantly, because our analysis is based on published case reports, the reported frequencies of symptoms, diagnostic findings, and treatment outcomes should not be interpreted as estimates of the true prevalence or natural history of ARCAPA. Case reports are inherently selective and tend to overrepresent unusual presentations and successful interventions, while asymptomatic or conservatively managed patients are likely underreported.

Unusual presenting scenarios in our cohort included atrial fibrillation with hemodynamic instability [4], cardiogenic shock [5], cardiac arrest during a marathon [6], worsening dyspnea in pregnancy [7], concurrent active tuberculosis [38], and association with a coronary fistula [39].

The physiological differences between ARCAPA and ALCAPA lie in the territory of myocardium supplied [2,3]. In ALCAPA, the left coronary system perfuses the majority of the left ventricle, leading to early severe ischemia [3,61]. In contrast, ARCAPA affects the right coronary territory, which is smaller and less critical for global systolic function [2,62]. These factors, together with the gradual decline in pulmonary artery pressure after infancy, allow for the development of extensive collaterals that often compensate for the steal phenomenon for decades [6,14,26]. However, long-term prognosis is not uniformly benign: progressive atherosclerosis in the collateral-supplying left coronary arteries, as seen in one fatal case in our series (1/59), can unmask critical ischemia [26]. Thus, while ARCAPA is generally associated with better survival into adulthood than ALCAPA, both anomalies warrant lifelong surveillance, particularly as patients age and acquire conventional cardiovascular risk factors [61,63].

The high proportion of incidental ARCAPA diagnoses (35.6% [21/59]) raises an important clinical question: how should an asymptomatic patient with incidentally detected ARCAPA be managed? In our analysis, such patients were often referred for surgical correction (54.2% [32/59] of all patients), but in 40.7% (24/59) of cases conservative management was chosen. Based on our data, the decision was largely driven by age, comorbidity burden, and the absence of objective signs of myocardial ischemia on stress testing. However, the absence of symptoms does not guarantee the absence of risk, as demonstrated by a fatal case of sudden cardiac death in an unoperated patient [26]. This underscores the importance of thorough work-up, including non-invasive ischemia testing (stress MRI, myocardial perfusion SPECT), before deciding on conservative management.

In our analysis, CTA was the most frequently used diagnostic modality (67.8% [40/59]), followed by coronary angiography (49.2% [29/59]). In our series, CTA was particularly valuable for preoperative planning in patients undergoing reimplantation of the RCA into the ascending aorta. According to ESC 2020 and ACC/AHA 2025 guidelines, CTA is the preferred first-line anatomical test for suspected ARCAPA because it provides high-resolution three-dimensional depiction of the anomalous vessel origin and course, which is essential for surgical planning [61,63]. Echocardiography retains an important role as a screening method, allowing suspicion of the anomaly based on indirect signs (dilation and tortuosity of the coronary arteries, pathological blood flow in the PA), but its direct diagnostic value is limited (6.8% [4/59]) [20,50,59]. According to guidelines, when CTA is not available or contraindicated, magnetic resonance angiography can be used as an alternative [61,63]. Invasive coronary angiography, according to current guidelines, is increasingly reserved for cases where concomitant coronary artery disease is suspected or when hemodynamic assessment of the shunt (Qp:Qs) is required [61,63].

In our series, the pulmonary-to-systemic flow ratio (Qp:Qs) was measured in only 10.2% (6/59) of cases. Our data show that Qp:Qs ranged from 1.2 to 1.38, yet management decisions varied. Based on our analysis and current guidelines, this parameter should be considered ancillary rather than decisive, and surgical indications should be based on symptoms, documented ischemia, and overall clinical assessment [61,63].

In our analysis, reimplantation of the RCA into the ascending aorta was the most commonly performed surgical technique (78.1% [25/32] of operated patients; 42.4% [25/59] of all patients). This approach is physiologically optimal because it eliminates the coronary steal phenomenon and establishes a dual-coronary system. In contrast, simple ligation of the anomalous RCA (6.3% [2/32] of operated patients; 3.4% [2/59] of all patients) was reserved for cases with prohibitively high risk of cardiopulmonary bypass. Ligation with concomitant CABG (15.6% [5/32] of operated patients; 8.5% [5/59] of all patients) was used when direct reimplantation was technically challenging.

In our series, one patient developed RCA thrombosis 17 years after surgery, combined with stenosis of the left circumflex artery [42]. The authors of that report noted that the anomalous RCA often has a thin, vein-like wall that does not normalize in diameter after correction. Based on our analysis and current guidelines, operated patients should undergo lifelong follow-up, including periodic imaging (CTA or MRI) and functional testing (stress echocardiography or myocardial perfusion SPECT) [61,63].

In our analysis, surgical correction (54.2% [32/59]) yielded favorable outcomes with symptom regression and improved perfusion [15,20,21,34,36,44,51,55,60], while conservative management (40.7% [24/59]) was chosen. Among the 24 conservatively managed patients in our series, the reasons were: high surgical risk in 29.2% (7/24), patient refusal in 50.0% (12/24), and treatment under consideration in 20.8% (5/24). According to ESC 2020 (Class I, Level C) and ACC/AHA 2025 (Class I, Level B-NR) guidelines, surgical correction is recommended in symptomatic patients or those with documented ischemia [61,63]. According to current guidelines, conservative management is acceptable in truly asymptomatic patients without evidence of ischemia, but regular follow-up is recommended [61,63].

Finally, it must be emphasized that all frequency data presented in this review are derived from published case reports and case series. As such, they are subject to publication bias and should not be used as prevalence estimates or as direct measures of treatment efficacy. They provide a descriptive overview of what has been reported in the literature, rather than a quantitative assessment of the true clinical spectrum or outcomes of ARCAPA.

### 4.1. Key Principles for the Management of Suspected ARCAPA

Based on current clinical guidelines (ESC 2020 [61], Russian guidelines 2024 [64], ACC/AHA 2025 [63]), we propose the following practical approach:

Clinical suspicion: ARCAPA should be considered in adults with unexplained chest pain, dyspnea, or exercise intolerance, particularly when echocardiography reveals dilated or tortuous coronary arteries or abnormal flow in the pulmonary artery [61,63].

Diagnostic confirmation: According to ESC 2020 and ACC/AHA 2025 guidelines, CTA is the preferred modality for definitive anatomical diagnosis and preoperative planning [61,63]. When CTA is contraindicated, magnetic resonance angiography may be used as an alternative.

Ischemia assessment: Current guidelines recommend functional testing (stress MRI, myocardial perfusion SPECT, or exercise stress testing) even in asymptomatic patients to document the presence or absence of myocardial ischemia before a conservative management decision is made [61,63].

Treatment decision: According to ESC 2020 (Class I, Level C) and ACC/AHA 2025 (Class I, Level B-NR), surgical correction (reimplantation of the RCA into the ascending aorta) is the treatment of choice for symptomatic patients or those with documented ischemia, ventricular dysfunction, or significant shunt [61,63]. Conservative management is acceptable in truly asymptomatic patients without evidence of ischemia, but regular follow-up with serial imaging and functional testing is recommended by current guidelines [61,63]. The Russian clinical guidelines (2024) similarly recommend restoration of a two-coronary system as the gold standard [64].

Lifelong follow-up: Both operated and conservatively managed patients should be considered for lifelong surveillance, as recommended by all major guidelines [61,63].

Multidisciplinary approach: According to ACC/AHA 2025 and ESC 2020, all treatment decisions should be made by a multidisciplinary heart team (cardiac surgeon, cardiologist, interventional cardiologist) with expertise in adult congenital heart disease [61,63].

### 4.2. Limitations

It is important to acknowledge that the majority of the included case reports and case series provided incomplete data, particularly regarding preoperative ischemia testing, long-term follow-up, and standardized definitions of high surgical risk. This limits the robustness of our pooled descriptive findings and underscores the need for cautious interpretation.

Our study has several limitations that should be considered when interpreting the results. Given the descriptive nature of the included studies, statements regarding the comparative performance of diagnostic modalities or the superiority of specific treatment strategies should be interpreted as observations derived from our case analysis rather than as definitive evidence-based conclusions. Whenever applicable, we have explicitly referenced published clinical guidelines to support management recommendations. First, the published case reports and case series included in our analysis were heterogeneous in terms of diagnostic work-up, follow-up duration, and reporting of clinical outcomes. This heterogeneity limits our ability to perform meta-analyses or to directly compare outcomes between surgical and conservative groups. Second, many reports did not provide detailed information on pre-operative ischemia testing (stress MRI, myocardial perfusion SPECT, exercise testing), which could influence the decision to operate. Third, the definition of “high surgical risk” varied across studies. Some authors considered advanced age alone as prohibitive, whereas others weighed specific comorbidities (e.g., end-stage renal disease, active malignancy, severe pulmonary hypertension). Fourth, long-term follow-up of conservatively managed patients was often incomplete or absent, making it difficult to assess the true rate of late adverse events in this group. Fifth, publication bias likely overrepresents cases with unusual presentations or complications, while truly asymptomatic patients diagnosed incidentally may be underrepresented. Consequently, the 35.6% rate of incidental findings in our analysis may actually be an overestimate of the true proportion in the general ARCAPA population. More broadly, all frequency data presented in this review should be interpreted as descriptive observations from the available literature rather than as estimates of the natural history of ARCAPA or the comparative effectiveness of different management strategies. The descriptive statistics summarize published experience rather than estimate population frequencies. Sixth, and most importantly, almost all published reports preferentially describe operated patients with favorable outcomes, which introduces substantial publication bias. Therefore, better outcomes after surgery cannot be interpreted as proof of superiority because patients selected for surgery differed substantially from those treated conservatively. This is a critical limitation that must be considered when interpreting our findings. Finally, the study is based on a retrospective analysis of published data, which inevitably carries the limitations inherent to any review of clinical cases. To address these problems, prospective registries with standardized data collection protocols are urgently needed.

### 4.3. Clinical Recommendations

Current guidelines support surgical correction for symptomatic ARCAPA or documented ischemia (ESC 2020 Class I, Level C; ACC/AHA 2025 Class I, Level B-NR) [61,63]. In asymptomatic patients without ischemia, conservative management is acceptable, but regular follow-up is recommended by current guidelines [61,63]. The Russian clinical guidelines (2024) similarly recommend restoration of a two-coronary system as the gold standard [64]. All major guidelines emphasize a multidisciplinary approach, particularly relevant for pregnancy planning [34,63].

Although descriptive comparisons between subgroups (symptomatic vs. asymptomatic, surgical vs. conservative) are presented in Table 1, formal statistical testing was not performed due to small sample sizes and incomplete data reporting.

## 5. Conclusions

ARCAPA, despite its rarity, is a clinically significant congenital anomaly that can first manifest in adulthood. Clinicians should consider ARCAPA in the differential diagnosis of adults with unexplained chest pain, dyspnea, or exercise intolerance, especially when non-invasive testing shows dilated or tortuous coronary arteries. When suspected, CTA appears to be the most effective confirmatory test. Functional ischemia testing should be considered in asymptomatic patients before choosing conservative management. The absence of pathognomonic symptoms highlights the importance of maintaining a high index of clinical suspicion and using modern imaging methods for timely diagnosis. CTA appears to be the preferred first-line anatomical imaging modality in contemporary clinical practice.

The management strategy for patients with ARCAPA requires an individualized approach that balances the potential risks of the natural course of the disease (including sudden cardiac death) against the risks of surgical intervention, especially in patients with comorbidities. Even after successful surgical correction, lifelong monitoring is recommended due to the possibility of long-term complications.

Future prospective multicenter registries are essential to better define the natural history of ARCAPA, identify which asymptomatic patients are at highest risk for sudden death, and establish evidence-based criteria for surgical versus conservative management. Until then, individualized decision-making by a multidisciplinary heart team remains the cornerstone of care.

## Figures and Tables

**Table 1:** Summary of demographic, clinical, diagnostic, treatment, and outcome data in 59 adult patients with ARCAPA (overall and by symptomatic status).

Parameter	Overall (*n* = 59)	Symptomatic (*n* = 46)	Asymptomatic (*n* = 13)
Demographics			
Male/Female, *n*	32/27	25/21	7/6
Median age (min–max), years	55 (18–80)	55.5 (18–77)	48 (18–80)
Clinical presentation			
Incidental finding, *n*/*N* (%)	21/59 (35.6%)	8/46 (17.4%)	13/13 (100%)
Chest pain, *n*/*N* (%)	28/59 (47.5%)	28/46 (60.9%)	0/13 (0%)
Dyspnea, *n*/*N* (%)	26/59 (44.1%)	26/46 (56.5%)	0/13 (0%)
Reduced exercise tolerance, *n*/*N* (%)	21/59 (35.6%)	21/46 (45.7%)	0/13 (0%)
Palpitations, *n*/*N* (%)	11/59 (18.6%)	11/46 (23.9%)	0/13 (0%)
Syncope, *n*/*N* (%)	2/59 (3.4%)	2/46 (4.3%)	0/13 (0%)
Laboratory data			
Troponin measured, *n*/*N* (%)	13/59 (22.0%)	11/46 (23.9%)	2/13 (15.4%)
–Elevated, *n*/*N* (%)	7/13 (53.8%)	7/11 (63.6%)	0/2 (0%)
–Normal, *n*/*N* (%)	6/13 (46.2%)	4/11 (36.4%)	2/2 (100%)
NT-proBNP measured, *n*	1	1	0
Diagnostic procedures			
ECG (available), *n*/*N* (%)	23/59 (39.0%)	18/46 (39.1%)	5/13 (38.5%)
–Normal, *n*/*N* (%)	8/23 (34.8%)	5/18 (27.8%)	3/5 (60.0%)
–Abnormal, *n*/*N* (%)	15/23 (65.2%)	13/18 (72.2%)	2/5 (40.0%)
Echocardiography (available), *n*/*N* (%)	30/59 (50.8%)	22/46 (47.8%)	8/13 (61.5%)
–Abnormal flow/tortuous vessels, *n*/*N* (%)	23/30 (76.7%)	19/22 (86.4%)	4/8 (50.0%)
–Normal, *n*/*N* (%)	7/30 (23.3%)	3/22 (13.6%)	4/8 (50.0%)
Imaging			
CTA performed, *n*/*N* (%)	40/59 (67.8%)	30/46 (65.2%)	10/13 (76.9%)
CAG confirming ARCAPA, *n*/*N* (%)	29/59 (49.2%)	26/46 (56.5%)	3/13 (23.1%)
MRI performed, *n*/*N* (%)	7/59 (11.9%)	7/46 (15.2%)	0/13 (0%)
SPECT performed, *n*/*N* (%)	5/59 (8.5%)	4/46 (8.7%)	1/13 (7.7%)
Cardiac catheterization with Qp:Qs, *n*/*N* (%)	6/59 (10.2%)	5/46 (10.9%)	1/13 (7.7%)
Treatment			
Surgical correction, *n*/*N* (%)	32/59 (54.2%)	25/46 (54.3%)	7/13 (53.8%)
–RCA reimplantation, *n*/*N* (%)	25/32 (78.1%)	20/25 (80.0%)	5/7 (71.4%)
–CABG with RCA ligation, *n*/*N* (%)	5/32 (15.6%)	4/25 (16.0%)	1/7 (14.3%)
–Simple RCA ligation, *n*/*N* (%)	2/32 (6.3%)	1/25 (4.0%)	1/7 (14.3%)
Conservative management, *n*/*N* (%)	24/59 (40.7%)	19/46 (41.3%)	5/13 (38.5%)
–High surgical risk, *n*/*N* (%)	7/24 (29.2%)	6/19 (31.6%)	1/5 (20.0%)
–Patient refusal, *n*/*N* (%)	12/24 (50.0%)	8/19 (42.1%)	3/5 (60.0%)
–Treatment under consideration, *n*/*N* (%)	5/24 (20.8%)	5/19 (26.3%)	0/5 (0%)
Treatment data not available, *n*/*N* (%)	2/59 (3.4%)	1/46 (2.2%)	1/13 (7.7%)
No treatment/died before treatment, *n*/*N* (%)	1/59 (1.7%)	1/46 (2.2%)	0/13 (0%)
Outcomes			
Patients with follow-up after surgery, *n*/*N* (%)	12/32 (37.5%)	8/25 (32.0%)	4/7 (57.1%)
Favorable outcome among followed patients, *n*/*N* (%)	12/12 (100%)	8/8 (100%)	4/4 (100%)
RCA thrombosis (late), *n*	1	1	—
No recurrence at 10 years, *n*	1	1	—
Sudden cardiac death (unoperated), *n*	1	1	—
No symptoms on medical therapy, *n*	6	—	6

Data are presented as *n*/*N* (%), median (min–max), or absolute numbers. Percentages for demographics, clinical presentation, diagnostic procedures, and main treatment categories are based on the total number of patients in each group. Percentages for subcategories (e.g., ECG, echocardiography findings, troponin levels, surgical techniques, reasons for conservative management, and favorable outcomes) are calculated based on the number of patients with available data or the relevant subgroup (e.g., those who underwent surgery or conservative management). ‘—’ indicates not separately analyzed, not applicable, or no events. Abbreviations: ECG, electrocardiography; NT-proBNP, N-terminal pro-brain natriuretic peptide; CTA, computed tomography angiography; CAG, coronary angiography; MRI, magnetic resonance imaging; SPECT, single-photon emission computed tomography; Qp:Qs, pulmonary-to-systemic flow ratio; RCA, right coronary artery; CABG, coronary artery bypass grafting; *n*/*N*, number of patients with the finding/total number in the group; min–max, range.

## Data Availability

Data sharing is not applicable to this article as no new data were created or analyzed in this study. All data presented in this analysis are derived from previously published case reports and case series, which are cited in the reference list.

## Abbreviations

The following abbreviations are used in this manuscript:

ARCAPA	Anomalous origin of the right coronary artery from the pulmonary artery
ALCAPA	Anomalous origin of the left coronary artery from the pulmonary artery
RCA	Right coronary artery
LCA	Left coronary artery
PA	Pulmonary artery
HF	Heart failure
CTA	Computed tomography angiography
CAG	Coronary angiography
MRI	Magnetic resonance imaging
SPECT	Single-photon emission computed tomography (myocardial perfusion scintigraphy)
CABG	Coronary artery bypass grafting
LAD	Left anterior descending artery
VACTERL	Vertebral anomalies, anal atresia, cardiac defects, tracheoesophageal fistula, esophageal atresia, renal anomalies, limb abnormalities
ESC	European Society of Cardiology
ACC/AHA	American College of Cardiology/American Heart Association
Qp:Qs	Pulmonary to systemic flow ratio (shunt magnitude)
